# *"PolyMin"*: software for identification of the minimum number of polymorphisms required for haplotype and genotype differentiation

**DOI:** 10.1186/1471-2105-10-176

**Published:** 2009-06-10

**Authors:** Ursula K Frei, Bernd Wollenweber, Thomas Lübberstedt

**Affiliations:** 1 Iowa State University, Department of Agronomy, Agronomy Hall, 100 Osborn Drive Ames, Iowa, 50011-1010, USA; 2 Aarhus University, Faculty of Agricultural Sciences, Department of Genetics and Biotechnology, Forsøgsvej 1, DK-4200 Slagelse, Denmark

## Abstract

**Background:**

Analysis of allelic variation for relevant genes and monitoring chromosome segment transmission during selection are important approaches in plant breeding and ecology. To minimize the number of required molecular markers for this purpose is crucial due to cost and time constraints. To date, software for identification of the minimum number of required markers has been optimized for human genetics and is only partly matching the needs of plant scientists and breeders. In addition, different software packages with insufficient interoperability need to be combined to extract this information from available allele sequence data, resulting in an error-prone multi-step process of data handling.

**Results:**

*PolyMin*, a computer program combining the detection of a minimum set of single nucleotide polymorphisms (SNPs) and/or insertions/deletions (INDELs) necessary for allele differentiation with the subsequent genotype differentiation in plant populations has been developed. Its efficiency in finding minimum sets of polymorphisms is comparable to other available program packages.

**Conclusion:**

A computer program detecting the minimum number of SNPs for haplotype discrimination and subsequent genotype differentiation has been developed, and its performance compared to other relevant software. The main advantages of *PolyMin*, especially for plant scientists, is the integration of procedures from sequence analysis to polymorphism selection within a single program, including both haplotype and genotype differentiation.

## Background

Many important agricultural traits are quantitatively inherited. Their phenotypes result from expression of multiple genes influenced by the environment. One approach to detect these genes is the candidate gene approach [1]. The definition of genes as 'candidate genes' can be based on the knowledge of their biochemical or physiological properties, homology to genes characterized in other species, or due to their close proximity on linkage maps to loci controlling the trait of interest. During the past decade, allelic sequence variation has been characterized for an increasing number of genes, and related to phenotypic variation of agronomic traits [2]. For example, Thornsberry [3] analysed the allelic sequences of *Dwarf8 *(d8) in 92 maize inbred lines and identified polymorphism associated with flowering time. Remington [4] studied the allelic sequences of 6 genes considered as candidate genes for variation in plant height and/or flowering time in a set of 102 maize lines, representing a broad cross section of the maize breeding germplasm. The ability to trace respective alleles affecting flowering by marker-assisted backcrossing might be useful to adapt germplasm to other latitudes.

Allelic variation within candidate genes is due to insertions/deletions (INDELs) or single nucleotide polymorphisms (SNPs). Of these, SNPs are the most abundant form of genetic variation, and different techniques for their efficient detection have been developed. Due to their shared history of mutation and recombination the polymorphisms found in candidate gene alleles will not segregate independently but show a correlation, called linkage disequilibrium (LD) [5]. The information content for allele differentiation of physically adjacent polymorphisms can, therefore, be redundant. Thus, the existing LD across a DNA sequence for a given germplasm of interest influences the density of marker coverage needed for allele differentiation. LD studies in maize revealed that the distance, over which LD decays, varies considerably depending on the population under examination. The narrower the germplasm base of lines examined, the higher the level of LD [4,6-8]. Genome wide analyses of LD in humans lead to the definition of haplotype blocks, i.e., chromosome regions of strong LD, versus recombination hot spots [9]. Haplotype blocks reflect the properties and history of that population, e.g., a small population size, a bottleneck leading to genetic drift, or population admixture. Similarities in LD patterns in different populations can be used to make assumptions about common ancestry. For example, similarities in LD can be due to the same ancient event, which occurred before separation of common ancestors of the populations [10]. Within a haplotype block only a few common haplotypes of limited diversity occur, which can be characterized by a small number of SNPs, also called haplotype-tagging SNPs (htSNPs).

In contrast to human genetics, plant breeders have the opportunity to generate large populations and expose them to various selection pressures. Due to the limited size of candidate genes in the range of few kb, and a very low chance of intragenic recombination events, the initial haplotype alleles for the individual candidate genes can be considered as fixed over few cycles of selection. In this situation, it is sufficient to select a minimum set of SNPs for the differentiation of those alleles, present in founder genotypes or in early generations, and to trace respective haplotypes or haplotype combinations ("diplotypes") in the offspring [11].

Various algorithms have been developed to find the minimum number of htSNPs based on haplotype block structure [12-17]. For the processing of allele sequence data and selection of minimum sets of SNPs, software packages are available for the different steps in this process, namely (1) allele sequence alignment [18-20], (2) identification of polymorphisms [21], and (3) selection of informative polymorphisms for allele differentiation [16,17]. However, the interoperability of these different software packages is insufficient, resulting in a cumbersome and error-prone multi-step process in assembling and analysing the data.

Our objective, therefore, was to develop software, which combines all steps required for selection of a minimal set of polymorphisms for genotype differentiation in offspring populations within a single program (*PolyMin*), starting from sequence alignments. Several examples of candidate gene alleles have been employed to compare the performance of *PolyMin *with BEST [17], for the central step of finding optimized SNP combinations for haplotype differentiation. Other programs such as SNPtagger [16] can only be used for some applications, as they do not tolerate more than two different bases per SNP site. None of the existing programs provides the possibility of differentiation of genotypes in a heterogeneous population including heterozygous individuals, derived from a set of known haplotypes, an important aspect in plant breeding programs. This step is included in *PolyMin*.

## Implementation

*PolyMin *has been developed using the C++ programming language and compiled with the C++ Builder 2009 compiler (CodeGear, Embarcadero Technologies, ScottsValley, California, USA) for the Microsoft Windows environment and is available from the website of the project . It has been tested on the Windows XP and Vista operating systems. A version compiled with the Qt SDK cross-platform development environment  is under development, which will enable *PolyMin *to run under Windows, Linux and Mac desktop operating systems.

The input file is the multiple sequence alignment in CLUSTAL format [19] of the candidate gene sequences from a set of genotypes as generated by, e.g., .

In a first step, the program identifies the different alleles (haplotypes) present in a set of genotypes and their frequency, based on sequence polymorphisms (INDELs and SNPs). *PolyMin *considers all polymorphisms found after the first base in common over all sequences. Further calculations are based on a haplotype matrix, where each haplotype occurs only once.

The algorithm of *PolyMin *starts with the whole set of polymorphisms and reduces them, while searching for the minimum number of polymorphisms. Optionally, INDELs, or singletons can be excluded. *PolyMin *then searches for polymorphisms that assign haplotypes to the same subgroups. In the following analysis *PolyMin *chooses always the first polymorphism of a certain pattern, in order to represent all redundant polymorphisms of the same pattern. Subsequently, a minimum set of polymorphisms, necessary to differentiate all – or as many as possible – haplotypes is established.

**Figure 1:**
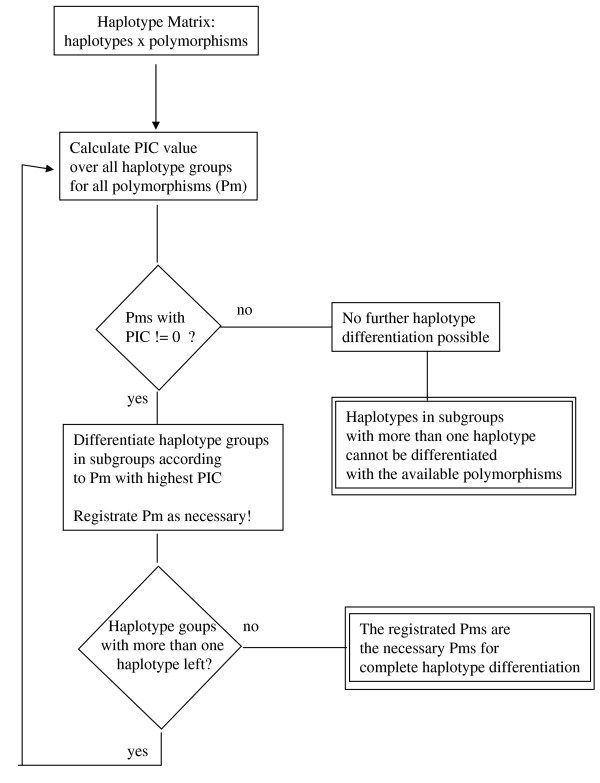
**Flowchart of haplotype differentiation (PolyMin-phaseI)**. Starting with the complete matrix of haplotypes × polymorphisms, the algorithm used in PolyMin divides the haplotypes in groups and subgroups, until no further subdivision is possible. As long as there are polymorphisms within a subgroup that have a PIC value different from 0, new subgroups can be formed. The process stops either if there are no more polymorphisms with PIC values different from 0 or if all haplotypes are divided into different subgroups. The results of the differentiation are recorded (see double framed boxes). Pm: polymorphism, PIC: polymorphism information content.

*PolyMin *performs polymorphism reductions either in one (phase I) or two (phase I+II) steps as described below. Initially, the polymorphisms for haplotype differentiation are selected in a cyclic procedure, based on their polymorphic information content (PIC) [22]:



Here, f_i _is the frequency of the i^th ^allele (Fig. 1). For each cycle the PIC value over all haplotype groups is calculated for all polymorphisms. The polymorphism with the highest PIC score is then selected for the next round of haplotype differentiation, generating new haplotype subgroups. The process stops when all haplotypes are differentiated, or when no polymorphisms are left in the subgroups (all PIC values become 0). A reduced set of polymorphisms for haplotype differentiation is thus identified (phase I).

This reduced set of polymorphisms can eventually be further minimized in a second step (phase II). By excluding single polymorphisms identified in phase I, their contribution on the differentiation of haplotypes is analyzed: if the number of different haplotypes remains identical, the respective polymorphism does not contribute to differentiation in addition to the other selected markers. These polymorphisms are then labelled as non-informative (phase II).

Finally, *PolyMin *identifies the polymorphisms necessary for differentiation of genotypes in the offspring, based on the whole data set or on a user-based selection of parental genotypes or alleles. The subsequent differentiation of the genotypes can be conducted either with the reduced set of polymorphisms or with the whole set of non-redundant polymorphisms. The expected marker haplotypes for all possible genotypes in the offspring, as well as those genotypes that cannot be distinguished in the data set, are displayed and can be used as basis for interpretation of SNP results in the offspring.

## Results

**Figure 2:**
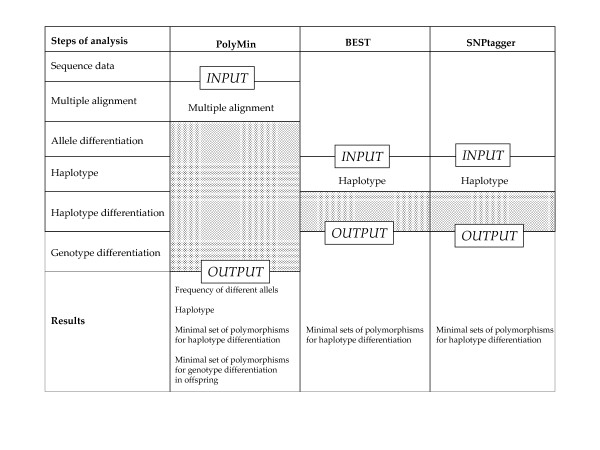
**Comparison of programs (the shaded areas show the steps of the analysis covered by the respective program)**.

To date there are no comparable programs which include all described steps from the detection of minimum number of SNPs to genotype differentiation in the offspring. Only single steps can be compared to other programs (see Fig. 2). Starting with a haplotype matrix, both BEST [17] and SNPtagger [16] select the minimum sets of SNPs for haplotype differentiation. Both software packages perform a strict reduction of polymorphisms. Of the 910 polymorphic base positions analyzed in the examples of Table 1, one third were SNPs (4.7% tri allelic) and two thirds were insertions/deletions. 20% of the insertions/deletions were combined with a SNP (5% tri allelic). A main drawback of SNPtagger is that it only allows two alleles per polymorphism, whereas BEST tolerates tri allelic SNPs as well as insertions and deletions.

**Table 1:** Comparison of *PolyMin *phase I, *PolyMin *phase I+II and BEST in their ability to detect minimum sets of polymorphisms and the usability of these polymorphism sets for genotype differentiation.

Gene	Number of different haplotypes	Number of possible genotypes	Program used	Minimum number of polymorphisms	Identified genotypes	Number of non- identifiable genotypes	Number of genotype groups left
CCoAOMT- 1	12	78	A^1^	6	67	11	5
			B^2^	5	29	49	21
			C^3^	6	73*	5*	2*
			D^4^	17	77	2	1

CCoAOMT-2	16	136	A^1^	7	128	8	4
			B^2^	5	49	87	25
			C^3^	6	110*	26*	12*
			D^4^	43	136	0	0

COMT	30	465	A^1^	12	447	18	9
			B^2^	8	234	231	75
			C^3^	9	415*	0	24*
			D^4^	74	465	50*	0

Lac1	16	136	A^1^	8	122	14	7
			B^2^	4	19	117	16
			C^3^	8	124*	12*	6*
			D^4^	27	132	4	2

Lac5-4	20	210	A^1^	9	182	28	13
			B^2^	6	51	169	45
			C^3^	9	182*	28*	13*
			D^4^	47	203	7	3

Lac5-6	8	36	A^1^	6	34	2	1
			B^2^	6	34	2	1
			C^3^	6	34*	2*	1*
			D^4^	7	34	2	1

Ra1	12	78	A^1^	11	78	0	0
			B^2^	11	78	0	0
			C^3^	11	78*	0	0
			D^4^	11	78	0	0

A^1 ^*PolyMin *phase I,

B^2 ^*PolyMin *phase I+II (= non-informative polymorphisms excluded),

C^3 ^BEST,

D^4^*PolyMin *genotype differentiation optimized (= all non-redundant polymorphisms included),

*The genotype differentiation for the minimum polymorphism set generated by BEST was performed with *PolyMin*.

Example data sets where extracted from NCBI (CCoAOMT- 1: AY323241–AY323271, CCoAOMT- 2: AY279004–AY279035, COMT: AY323272–AY323305, Lac1: AY464016–AY464051, Ra1 DQ013174–DQ013202) or from Xing et al. [25].

The algorithms of BEST and *PolyMin *exclude first all redundant polymorphisms (named *binary equivalent *in BEST), but differ in subsequent steps: BEST begins with single polymorphisms and adds further polymorphisms step by step according to its selection criteria. Avi-Itzhak thoroughly described an algorithm for minimum SNP detection [12], similar to the one used for *PolyMin*, whereby the SNPs are reduced in two phases. Avi-Itzak uses the Shannon Entropy [23] as a measure of haplotype diversity for his first phase of polymorphism reduction [12], whereas in *PolyMin *the PIC value was employed as selection criterion. An example for the second step of polymorphism reduction is presented in Table 2: the exclusion of polymorphisms 1 and 4 (shaded rows) does not result in a further reduction of the number of haplotypes (columns). Therefore, in this sample these polymorphisms can be omitted. *PolyMin *does not exclude these polymorphisms, when only phase I is applied. The minimum number of polymorphisms required for haplotype differentiation is, therefore, overestimated by phase I in *PolyMin*. However, these polymorphisms can be excluded in phase II and marked as non-informative. Taylor and Provart [24] apply a slightly different approach to determine the optimal sets of CAPS marker for genotyping compared to PolyMin, however the selection criteria, (Cost(set)) is similar to the PIC value: (Cost(set)) = 1-(PIC*number-of-individuals). A direct comparison of the two programs was not possible, as CapsID selectively analyses polymorphisms that alter restriction enzyme recognition sites. For the subsequent genotype differentiation the suboptimal selection of polymorphisms by *PolyMin*'s phase I turned out to be superior to those generated by *PolyMin *phase I + II, as more of the possible genotypes can be distinguished in the offspring. For an optimized differentiation of genotypes the initial set of non-redundant polymorphisms has to be used. In Table 1, various contrasting data sets were analysed, applying *PolyMin*-phase I (A) or *PolyMin*-phase I + II (B), and BEST (C) respectively. The maximal number of genotypes that can be differentiated in the data set is calculated on the basis of the initial non-redundant set of polymorphisms (D). Depending on the number of haplotypes present in a population, the number of possible genotypes is:



where g is the number of genotypes, and h the number of haplotypes. Compared to *PolyMin *phase I and phase I+II, BEST returns minimum polymorphism sets similar to phase I or stricter and never found fewer polymorphisms than *PolyMin *phase I+II for haplotype discrimination.

**Table 2:** Result from *PolyMin *phase I, showing 9 polymorphisms, selected for the differentiation of 13 haplotypes (reduced data set for demonstration).

				1	2	3	4	5	6	7	8	9	10	11	12	13
no	pm	Bp	rp	SEQ09	SEQ10	SEQ11	SEQ14	SEQ12	SEQ13	SEQ03	SEQ02	SEQ01	SEQ04	SEQ05	SEQ06	SEQ07 SEQ08

**1**	**7**	**8**	**1**	**-**	**-**	**-**	**-**	**-**	**-**	**A**	**A**	**A**	**A**	**A**	**A**	**A**

2	14	15	4	A	G	G	G	G	G	A	A	A	A	A	G	G

3	62	63	1	-	-	-	-	-	-	-	A	A	A	A	A	-

**4**	**3**	**4**	**9**	**C**	**A**	**A**	**C**	**C**	**C**	**C**	**C**	**C**	**C**	**C**	**C**	**C**

5	13	14	1	-	-	-	-	-	-	-	-	-	A	A	A	A

6	63	64	1	C	C	C	C	C	C	T	C	T	C	T	C	C

7	12	13	1	A	A	A	-	-	A	A	A	A	A	A	A	A

8	22	23	19	-	-	-	-	A	A	-	-	-	-	-	-	-

9	52	53	2	A	-	A	A	A	A	A	A	A	A	A	A	A

Lines in bold: polymorphisms that will be excluded from the set, when using *PolyMin *phase II, as they do not contribute to the differentiation of the 13 haplotypes.

no – polymorphism number,

pm – name of the polymorphism,

bp – position of the polymorphism within the sequence,

rp – number of redundant polymorphisms found in the whole sequence.

In order to compare the power of minimum polymorphism sets generated by BEST, the genotype differentiation was calculated with *PolyMin *based on the BEST results. The number of genotypes identified by the minimal polymorphism sets generated by *PolyMin *phase I and BEST are different (Table 1). As long as the number of polymorphisms per minimum polymorphism set is greater for phase I as in BEST, *PolyMin *phase I can identify more genotypes. In the case of CCoAOMT-1 and Lac1, the polymorphism sets generated by BEST distinguish more genotypes with the same number of polymorphisms than the one generated by *PolyMin *phase I, indicating that the algorithm used by BEST to minimize polymorphisms is superior to the procedure used in *PolyMin *in these two cases. The genotype differentiation with the initial set of non-redundant polymorphisms shows, that even when applying all polymorphisms, it will not be possible to differentiate all genotypes (for example in CCoAOMT-1, Lac1, and Lac5-6, Table 1).

SNPtagger developed for human genetics can only analyse two alleles per polymorphism. The program is, therefore, only of minor use in plant genetics. The main drawback of programs developed for human genetics is however the missing analysis of genotype differentiation, a fundamental aspect in plant breeding projects.

## Discussion

The *PolyMin *software was developed to differentiate candidate gene alleles in selected or natural populations and to discriminate genotypes in offspring with an optimal set of polymorphisms. The program is therefore of particular interest for plant geneticists and breeders. *PolyMin *was designed to handle both types of polymorphisms – SNPs and insertion/deletions – and has no restrictions as to the number of alleles at one polymorphic site. Polymorphisms with more than two alleles occur rather frequently in plants, especially in highly heterozygous individuals of out-breeding crops. On average 7.3% of the polymorphisms in the example alignments of Table 1 had more than two alleles. Most programs with similar objectives were developed for human genetics, where large fragments of chromosomes or whole genomes are analysed. This requires a preliminary subdivision of the fragments into haplotype blocks, i.e., regions with strong LD. Within those haplotype blocks, a wealth of polymorphisms are available for differentiation, making it necessary to develop algorithms for efficiently reducing the number of polymorphisms in order to obtain a maximum of information for given cost input. Respective results can be used to describe, e.g., the ancestry of different sub-populations with different susceptibilities to diseases or other traits of interest [10,14]. The main focuses in human genetics are retrospective analyses.

Comparable marker analyses in plant breeding focus mostly on future generations by shaping new populations in the process of marker-assisted selection. Typically, the number of meioses accumulated in elite germplasm is rather low, so that breeders typically work at the level of chromosome blocks with extended LD rather than single independent segregating genes.

## Conclusion

*PolyMin *provides plant breeders with the design of optimal marker assays to discriminate possible genotypes derived from these extended haplotype blocks and their expected frequency in the offspring, based on the parental genotypes selected. Polymorphisms selected by *PolyMin *facilitate comparison of actual genotype frequencies in populations with and without any selection pressure or under divergent selection regimes. It is planned to integrate the analysis of diplotyping results into *PolyMin *to highlight polymorphisms, which diverge from expectations.

## Availability and requirements

**Project name**: *PolyMin*

**Project home page**: 

**Operating system(s)**: Windows XP, Windows Vista.

**Programming language**: C++

**Other requirements**: none

**Licence**: free non-commercial research-use licence.

**Any restrictions to use by non-academics**: none.

## Authors' contributions

UKF developed the algorithm for the analysis and planned and supervised the manuscript. BW conceived the software, carried out its design and participated in writing the draft of the manuscript. TL participated in the design of the study and helped to draft the manuscript. All authors have read and approved the final manuscript.
